# Time-series analysis of *Campylobacter* incidence in Switzerland

**DOI:** 10.1017/S0950268814002738

**Published:** 2014-11-17

**Authors:** W. WEI, G. SCHÜPBACH, L. HELD

**Affiliations:** 1 Department of Biostatistics, Epidemiology, Biostatistics and Prevention Institute, University of Zurich, Switzerland; 2 Veterinary Public Health Institute, University of Bern, Switzerland

**Keywords:** Incidence of *Campylobacter* in humans, prevalence of *Campylobacter* in broilers, time-series analysis

## Abstract

Campylobacteriosis has been the most common food-associated notifiable infectious disease in Switzerland since 1995. Contact with and ingestion of raw or undercooked broilers are considered the dominant risk factors for infection. In this study, we investigated the temporal relationship between the disease incidence in humans and the prevalence of *Campylobacter* in broilers in Switzerland from 2008 to 2012. We use a time-series approach to describe the pattern of the disease by incorporating seasonal effects and autocorrelation. The analysis shows that prevalence of *Campylobacter* in broilers, with a 2-week lag, has a significant impact on disease incidence in humans. Therefore *Campylobacter* cases in humans can be partly explained by contagion through broiler meat. We also found a strong autoregressive effect in human illness, and a significant increase of illness during Christmas and New Year's holidays. In a final analysis, we corrected for the sampling error of prevalence in broilers and the results gave similar conclusions.

## INTRODUCTION

*Campylobacter* is a major foodborne pathogen causing campylobacteriosis in humans and is the most common bacterium that causes gastroenteritis worldwide [1]. The onset of disease symptoms usually occurs 2–5 days after infection, but can range from 1 to 10 days [2]. Since 2005, campylobacteriosis has been the most common food-associated notifiable illness in Switzerland. From 2005 to 2009 reports of campylobacteriosis cases increased up to 100 cases/100 000 inhabitants [3].

*Campylobacter* species are present in most warm-blooded animals and campylobacteriosis is transmitted to humans from animals or animal products. In animals, *Campylobacter* seldom causes disease. For humans, transmission occurs through consumption of contaminated food, water and milk products, as well as directly from animals and the environment [4, 5]. Contact with and the ingestion of raw or undercooked broiler meat are considered the dominant risk factors for infection [6]. The consumption of chicken is estimated to account for between 40% and 70% of human infections [7]. Several studies show that the incidence of *Campylobacter* colonization in broiler flocks and the incidence of campylobacteriosis in humans show a concordant seasonality in Europe [8, 9].

*Campylobacter* illness in humans has a clear seasonal pattern with an annual peak during the summer, which may be due to high temperatures encouraging increased opportunity to contact transmission sources, e.g. more outdoor activities, more barbecues with undercooked poultry meat. The prevalence of *Campylobacter* in broilers (prevalence in broilers) is also higher during the summer in Switzerland. Several studies show that increasing prevalence in broilers increases incidence in humans. However, the extent to which the human incidence increase can be explained by the increase in prevalence in broilers is unclear. In this study, we investigate the presence and strength of the relationship between prevalence in broilers and the incidence of *Campylobacter* illness in humans (incidence in humans).

## DATA

### Incidence in humans

**Fig. 1.**
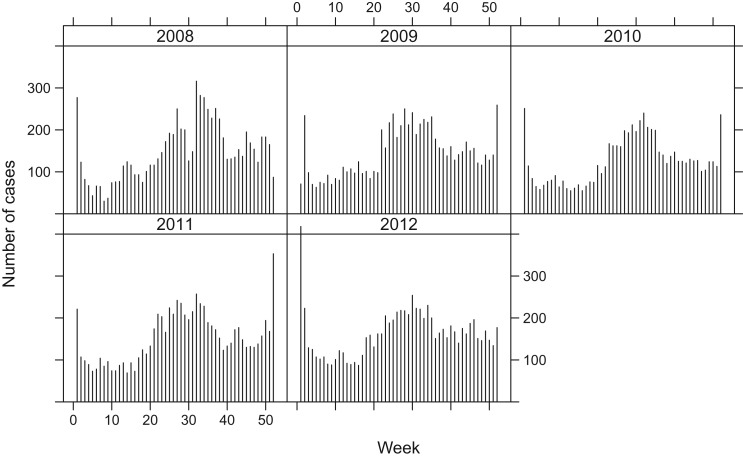
Weekly number of reported human campylobacteriosis cases in Switzerland, 2008–2012

Since a mandatory notification was introduced in 1988, all laboratory confirmations of *Campylobacter* samples of patients must be notified to the Federal Office of Public Health (FOPH) in Switzerland. Human samples were cultured for *Campylobacter* spp. in several different laboratories. The number of cases reported to FOPH as well as the notification rate are published weekly in the FOPH Bulletin [10]. To explore the disease dynamics, we aggregated the data to weekly counts of *Campylobacter* cases in 2008-2012, shown in Figure 1. The disease follows a seasonal pattern, with a peak every summer, and a second distinct peak of around 2–3 weeks during the Christmas holiday season, possibly related to the increased consumption of fondue Chinoise [10, 11]. When eating fondue, the risk of contamination increases when raw meat is placed on the same plate as the cooked food. This seasonal pattern is visible in all five years considered.

### Prevalence in broiler flocks

The prevalence in broilers is monitored by testing broiler samples, which are collected by private companies or the Swiss Federal Veterinary Office (SFVO) in Switzerland. In 2008–2009, the data were from the pre-delivery control system in a large slaughterhouse. A total of 1959 flocks were tested in 2008 and 1983 flocks in 2009. Calculation of prevalence was based on the date of slaughter, where a slaughter group was regarded as positive if at least one of the samples tested positive for *Campylobacter*. From 2010 to 2012, we calculated the prevalence based on the sample results from SFVO. In the surveillance system, a random sample of broiler flocks was investigated at slaughter every week. The broiler slaughter plants included in the surveillance programme account for >90% of the total production of broilers in Switzerland. The number of samples for each plant was determined in proportion to the number of animals slaughtered per year. Each sample represents one flock [3]. The poultry samples were all analysed in the Swiss reference laboratory (Centre for Zoonotic Diseases, Bacterial Diseases and Antimicrobial Resistance, University of Bern). Poultry flocks were sampled using five cloacal swabs per flock (each from five different broilers) in standard transportation medium (Transport swabs, Oxoid TS0001A, Amies W/O CH). At the laboratory, cloacal swabs were pooled and direct culture was carried out on selective medium suitable for *Campylobacter* (mCCDA, Oxoid, Switzerland). Samples were further identified to the species level, with *C. jejuni* and *C. coli* being the main species identified. In total, 489 samples were positive, 923 negative and 129 results were unavailable. Between 1 and 37 samples were tested each week. The prevalence based on the samples in week *t* is then calculated as 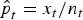, where *x*_*t*_ denotes the number of positive sampled groups and *n*_*t*_ the total number of samples.

However, in some weeks no samples were collected, i.e. *n*_*t*_ = 0. For example, this is often the case in weeks 1 and 52, which may be due to public holidays in Switzerland. We applied an algorithm to impute the missing prevalence values (see below). We also used this approach to adjust for the sampling error in the prevalence estimates.

**Fig. 2.**
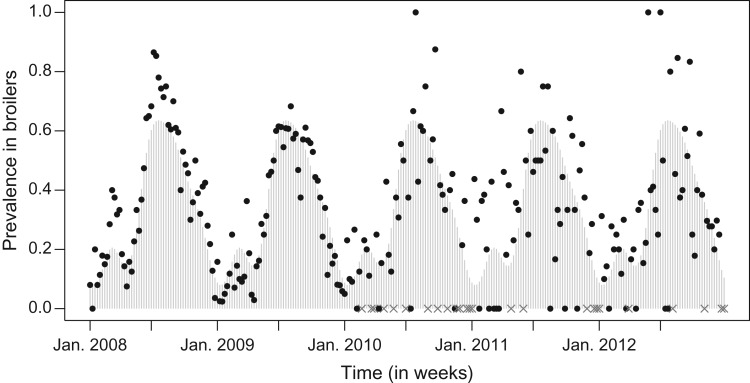
Prevalence in broilers. The observed prevalence is indicated by dots, the missing values by grey crosses at the x axis and the fitted values by light grey bars.

The prevalence of *Campylobacter* in broilers, has a similar seasonal pattern as incidence in humans. The prevalence pattern shows a gradual rise from the beginning of the year, peaking in summer, and decreasing in autumn, until the prevalence reaches the lowest value towards the end of the year, see Figure 2.

## METHODS

### Imputation of prevalence in broilers

To investigate the association between disease incidence in humans and prevalence in broilers, we first need to impute the prevalence in weeks where no samples have been taken. One possibility is to conduct a simple imputation, such as ‘observation carried forward’ or filling in the value of the same week from the last year. Alternatively a regression model can be used assuming that the prevalence follows a seasonal pattern, as suggested in several studies [8, 12]. A variance-stabilizing transformation is applied before fitting using the arcsine square-root transformation 
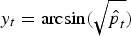
, where 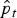 is the estimated prevalence in week *t*. This transformation is commonly used for proportions where the sampling distribution of *y*_*t*_ can be shown to have variance approximately equal to 1/(4*n*_*t*_). We now regress *y*_*t*_ on a superposition of sinusoidal waves [13] with frequencies *ω*_*s*_ = 2*πs*/52, (*s* = 1, … , *S*), i.e.

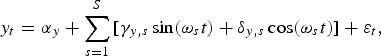

where *ε*_*t*_ ~ *N*(0,1/(4*n*_*t*_)). This formulation corrects for the sampling error of the prevalence estimates by including the inverse variances 4*n*_*t*_ as weights, giving more weight to prevalence estimates with a larger number of samples. The fitted values can subsequently be used to impute the missing prevalence in weeks without samples.

Models with a different number *S* of sinusoidal waves are listed in Table 1, where *S* = 4 can be identified as the best model according to Akaike's Information Criterion (AIC). The fitted model with *S* = 4 is shown in Figure 2, where the fitted values are shown in grey bars, the weeks without samples indicated by grey crosses and original values indicated by black dots. This model captures a high prevalence in the summer and a smaller peak in the spring.

**Table 1.** Analysis of imputation models on prevalence in broilers

*S*	*p*	Log *L*	AIC
1.	3	50·91	−93·83
2	5	54·69	−97·37
3	7	64·6	−113·19
**4**	**9**	**66·78**	**−113·56**
5	11	66·79	−109·59

AIC, Akaike's Information Criterion.

The log-likelihood is denoted as log *L, p* is the number of parameters in the model, *S* is the number of sinusoidal waves, AIC = −2 log *L* + 2*p*.

### Time-series model

We applied the time-series model for count data as described in [14] on the number of campylobacteriosis cases in humans. The formulation is based on an additive decomposition of disease incidence into an endemic component *X*_*t*_ and an autoregressive epidemic component *Y*_*t*_. Let *Z*_*t*_ denote the number of reported cases in week *t*, where *t* ∈ 1,2, … , *n*. The basic model assumes that *X*_*t*_ ~ Po(*ν*_*t*_) and *Y*_*t*_ ~ Po(*λ Z*_*t*−1_) are independent, so
(1)
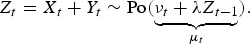

Here *X*_*t*_ represents the endemic component with Poisson rate *ν*_*t*_ where

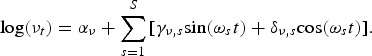

As in the previous section, the frequencies *ω*_*s*_ = 2*πs*/52 are used but the number *S* of sinusoidal waves may be chosen differently. The epidemic component *Y*_*t*_ forms an autoregression on the total number of cases *Z*_*t*−1_ in the previous week with unknown parameter *λ*. The Poisson assumption for *Z*_*t*_ ~ Po(*μ*_*t*_) can be relaxed using a negative binomial distribution NBin(*μ*_*t*_, *ψ*) to correct for possible overdispersion.

By using the approach described by [15], additional (time-changing) explanatory variables can be included in the endemic or epidemic component. The epidemic component is then time-dependent, i.e. *λ*_*t*_ replaces *λ* in equation (1). In our analysis, an indicator variable

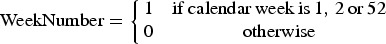

for the fondue season is included in the endemic component to adjust for the sharp incidence peak around the New Year period. In addition, the prevalence in broilers can be used as an explanatory variable either as the endemic (model A) or as the epidemic (model B) component:

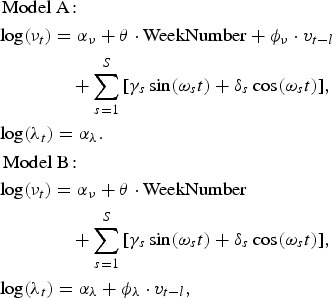

where *v*_*t*−*l*_ is the prevalence with *l* ∈ {−3,…,3} weeks of lag. All calculations were carried out in the statistical software environment R.3.1.1 [16] using the package surveillance 1.8.0 [14].

## RESULTS

### Temporal analysis excluding prevalence in broilers

We conducted a univariate analysis of incidence in humans, adjusting for seasonality and the effect of the New Year. The model without an autoregressive epidemic component is a generalized linear regression model (GLM) with a Poisson or negative binomial observation model. When including a seasonality adjustment, the negative binomial model fits much better in terms of AIC than the corresponding Poisson model. This indicates that the number of campylobacteriosis cases in humans is overdispersed. The inclusion of the epidemic component with estimated autoregressive parameter 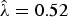 leads to a further improvement of the maximized log-likelihood and AIC. Consequently, the use of a GLM seems inappropriate for these data. Increasing the number of sinusoidal waves to *S* = 2 leads to a slight improvement of the maximum log-likelihood, therefore the best model in terms of AIC is the negative binomial model with two sinusoidal waves as well as an autoregression component.

**Fig. 3.**
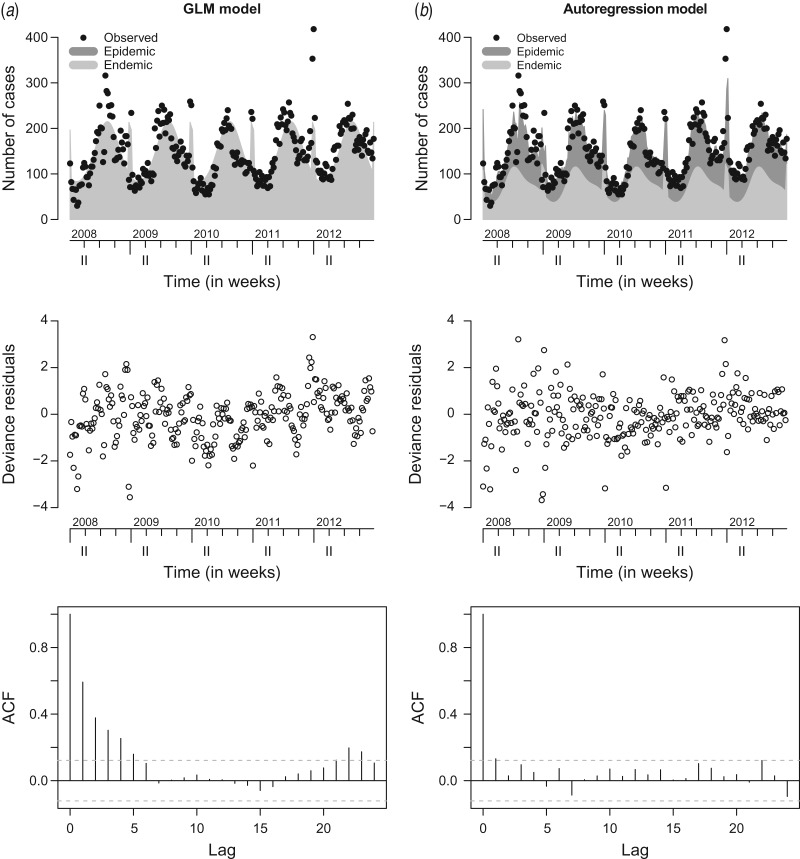
Observed and fitted number of cases, deviance residuals and autocorrelation function (ACF) in (*a*) the generalized linear model (GLM) and (*b*) the autoregression model.

Figure 3 compares the observed and fitted number of cases for the two final negative binomial models with and without an autoregression component. The fitted values are decomposed into the endemic and the epidemic components. Deviance residuals and the autocorrelation functions (ACF) of the residuals in the corresponding models are also shown in Figure 3. The residuals of the autoregressive model are approximately uncorrelated, while in the GLM the residual autocorrelation is substantial for small time lags, e.g. ACF = 0.6 for lag *l* = 1. This gives further evidence for the need to include the autoregressive component.

### Temporal analysis including prevalence in broilers

**Fig. 4.**
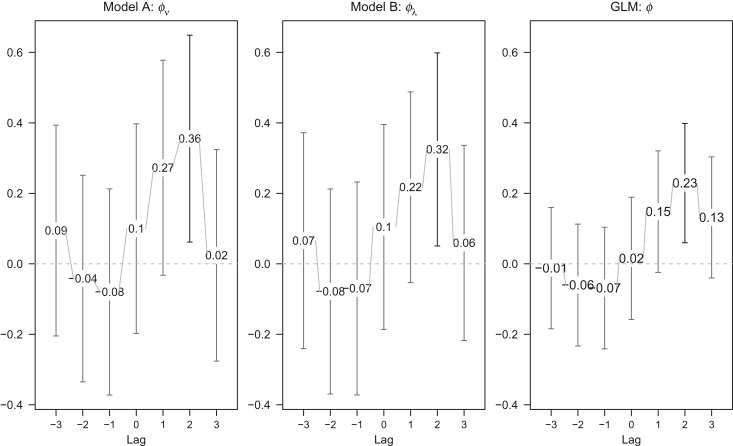
Estimated coefficient of the lagged prevalence in broilers (with 95% confidence intervals) in the endemic (model A) or epidemic (model B) component or generalized linear model (GLM) with seasonality (*S* = 2).

We further investigated the impact of the inclusion of prevalence in broilers as an explanatory variable in model. A delayed effect of prevalence in broilers on human illness should be taken into consideration, due to varying consumption periods of broiler meat (e.g. time for meat in transport, retail and consumption) and the incubation period of the illness. Therefore we investigated the impact of prevalence in broilers by varying lags up to 3 weeks. Forward lags *l* ∈ {−3,−2,−1} in models A and B were also included into the analysis to assess the direction of the association between prevalence in broilers and incidence in humans. Figure 4 gives the estimated coefficient 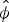 with its 95% confidence interval for each model. For comparison, results from a negative binomial GLM are also included.

The estimated effect is largest for lag *l* = 2 with 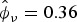 (*P* = 0·018), 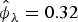 (*P* = 0·02) and 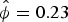 (*P* = 0·008), with 95% confidence intervals covering only positive values indicating a significant impact on incidence in humans. The evidence for a positive lag *l* = 1 association is weaker, since the lower limits of the 95% confidence interval are now negative. There is no evidence for an association for negative lags (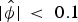 for all three models). This indicates that a change in the prevalence in broilers might be associated with a change in incidence rate in humans after a 2-week delay. The comparison of each model in terms of AIC values is shown in Table 2. In terms of AIC, including the prevalence in the previous 2 weeks in the endemic component gives the best fit (highlighted in Table 2).

**Table 2.** AIC values in models including the prevalence in broilers with different weeks of lag and seasonality S = 2

Lag	Model A	Model B	GLM
−3	2407·32	2407·54	2482·39
−2	2407·63	2407·42	2481·94
−1	2407·42	2407·5	2481·8
0	2407·27	2407·23	2482·38
1	2404·52	2405·3	2479·6
2	**2401·97**	2402·4	2475·48
3	2407·68	2407·53	2480·17

AIC, Akaike's Information Criterion.

The model with smallest AIC value is given in bold face.

Model A is a model with the prevalence in broilers as endemic component, model B with the prevalence as epidemic component. GLM represents a generalized linear model including prevalence without an autoregressive component.

**Fig. 5.**
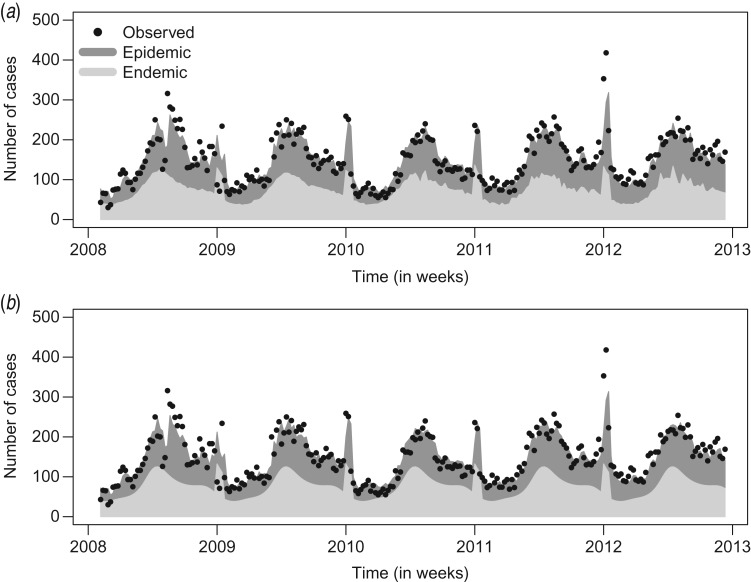
Fitted number of disease cases in humans in models with (*a*) the prevalence in broilers and (*b*) the prevalence after correction for sampling error as explanatory variable.

Additional analyses were performed to study the impact of sampling error in the estimates of prevalence in broilers. To do this, the fitted prevalence from the ‘Imputation of prevalence in broilers’ sub-section has been used instead of the original values. The estimated coefficient 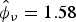 (s.e. = 0·59) of the prevalence in broilers 2 weeks earlier is now larger than it is in Figure 4 (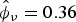), as expected after correction for sampling error. The corresponding AIC value from the best model with *S* = 2 and lag 2 is 2400·56, which is slightly better than the best model A with original values. Figure 5 compares the fitted number of disease cases from the original and estimated prevalence in broilers, respectively, which are quite similar. This analysis shows that after correction for sampling error in the prevalence estimates of broilers, a stronger effect of prevalence in broilers can be observed.

## DISCUSSION

In the present paper, we applied a time-series model for incidence of *Campylobacter* in humans in order to investigate a possible association between campylobacteriosis in humans and prevalence of *Campylobacter* in broilers. We found a positive direct association with a delay of 2 weeks. The prevalence in broilers in the previous 2 weeks is shown to have a significant association with illness in humans, indicating that human *Campylobacter* cases are partly explained by contagion through broiler meat.

A particular feature of the proposed model is the decomposition of the disease incidence into an endemic and an epidemic component. Compared to a standard GLM, our formulation is able to account for autocorrelation by including an autoregressive component. This turns out to be important for the spread of infectious diseases in animals or humans.

There are some limitations to our study. Only laboratory-confirmed cases of illness are reported to the national surveillance data, those reported data may not be representative of all campylobacteriosis infections. However, given the lack of human infection data collection, these data are the best source of information available. Only cases with laboratory confirmation were included, and we are well aware of the problem of possible underreporting in the time series of campylobacteriosis cases [6]. On the other hand, our formulation can be considered as useful as long as the underreporting rate does not change with time [15]. The human data comprise cases of all ages. Thus, difference of broiler consumption patterns in different age groups may lead to a shift of the evaluation on the impact of broilers, but it is impossible to discern such shifts from age-aggregated surveillance data.

Human incidence may not only be related to the presence of *Campylobacter* spp. on poultry carcases, but also on the number of bacteria present on each contaminated carcass. However, because the laboratory method for enumeration of *Campylobacter* is quite time-consuming, few data are available on seasonal variation of *Campylobacter* counts on poultry. A study in Belgium found no seasonal variation in *Campylobacter* counts [17].

There are many samples (68·4%) of broiler meat without information about the specific *Campylobacter* species, which restricts us to derive prevalence from either *C. jejuni* or *C. coli*, the two species associated with most of the disease cases. We are well aware that the data of prevalence in broilers originating from different sources for the years 2008–2009 and 2010–2012, respectively. But the seasonal pattern shows few differences between the two data sources. In the data which only originate from one large slaughterhouse, the prevalence during winter time is slightly lower compared to the data representing the whole population. This might be due to the fact that this slaughterhouse has a comparably large percentage of flocks participating in label programmes for ‘animal friendly’ housing. These animals originate from stables with outdoor access or a ‘winter garden’. Temperatures in these non-isolated poultry houses may be lower during winter than in traditional indoor housing. In addition, there is uncertainty about the true prevalence in broilers, when obtained from the collected sample results. To adjust for this, we conducted an analysis based on the fitted prevalence values from the imputation models. After adjusting for the sampling error in the whole prevalence series, the prevalence imputed shows a quite similar pattern to the prevalence in 2008–2009, which indicates that the sample bias from one slaughterhouse is not a distinct problem. The fitting results are slightly better in terms of AIC and the estimated impact of prevalence in broilers is much larger, which gives further evidence that the prevalence in broilers plays an important role for human infections.
